# Editorial: Multi-scale urban built environment and human health

**DOI:** 10.3389/fpubh.2025.1719990

**Published:** 2025-10-29

**Authors:** Xin Li, Ye Liu, Yuliang Zou, Chen Zhong

**Affiliations:** ^1^Guangxi University, College of Civil Engineering and Architecture, Nanning, China; ^2^Sun Yat-sen University, School of Geography and Planning, Guangzhou, China; ^3^Wuhan University, School of Public Health, Wuhan, China; ^4^University College London, The Bartlett Centre for Advanced Spatial Analysis, London, United Kingdom

**Keywords:** multi-scale analysis, built environment (BE), settlement, public health, big data

## 1 Introduction

Urbanization not only reshape where people live and how move, but also reshape the patterns of public health. The contributions in this Research Topic show that the built environment (BE) exerts health effects across scales, from housing quality and street-level greenness to neighborhood services and citywide mobility systems. Collectively, they move beyond single-exposure, single-scale perspectives toward scale-aware, pathway-specific, and context-sensitive understandings of how place affects health. Based on the submitted manuscripts, we can observe three insights that matter in shaping health issues: (1) measurement refers to exposure definitions, spatial and temporal scales, and interpretability choices that influence inferences; (2) social pathways refers to community attachment, cohesion, and affordability that shape mental and cardiometabolic outcomes alongside physical features; and (3) policy levers exist at multiple levels, i.e., parcel, street, neighborhood, and city, enabling coordinated, equity-centered action that captures co-benefits while managing trade-offs.

## 2 The multi-scale imperative

Understanding the health impacts of urban environments requires moving beyond single-scale analyses. The BE factors operate simultaneously across multiple spatial scales, e.g., dwelling unit, neighborhood, district, and metropolitan region. Each scale presents distinct exposure pathways and intervention opportunities. The contributions in this Research Topic demonstrate that scale matters profoundly, as a fundamental determinant of how environmental factors translate into health outcomes.

Three works exemplify this multi-scale approach through innovative methodologies. Peng et al. employ machine learning to uncover non-linear threshold effects of BE characteristics on weekend metro ridership in Shanghai, revealing that land use mixture, distance to CBD, and transit connectivity operate through distinct spatial ranges with specific optimal thresholds. The findings challenge linear assumptions pervading transportation-health research, showing that maximum ridership occurs when land use entropy remains below 0.7 and bus line density reaches 35 routes. Similarly, to match cooling service provision with resident needs, Song et al. develop a supply-demand framework to evaluate park cold island effects across neighborhoods, integrating remote sensing data with Thiessen polygon analysis. The finding reveals that 40% units exhibit supply-demand mismatches, concentrated in areas with complex building environments and poor landscape connectivity. Besides, He et al. investigate the relationship between housing quality and tenant mental health across seven urban villages in Shenzhen, as demonstrates how community-level attachment mediates between dwelling conditions and psychological wellbeing, and illustrates how individual, household, and neighborhood scales interact to shape health outcomes.

## 3 Bridging environmental metrics and health pathways

A common theme across these works is the imperative to elucidate the behavioral, social, and physiological pathways linking environments to health. Several works employ structural equation modeling approaches to unpack sophisticated mediation mechanisms. For instance, Zuo et al. examine how urban green space influences older adults” mental health through relative deprivation, physical activity, and social trust. The findings reveal that green space coverage reduces perceived relative deprivation and promotes physical activity, which in turn enhance mental health. It suggests that environmental equity and behavioral activation represent more proximate pathways than social cohesion for this population. Feng and Zheng's study toward Nanjing older adults uncovers significant gender differences in how BE affects subjective wellbeing, with community-level factors explaining 33.68% of variance for female vs. 28.50% for male. Household structure operates through gendered divisions of domestic labor and culturally-specific norms around filial piety, demonstrating how socio-cultural mechanisms intersect with physical environments. Gu et al. trace the pathways from BE through health behaviors to hypertension risk, revealing that clinic density, supermarket proliferation, and road network characteristics influence disease risk by shaping residents' walking time, physical activity duration, and fruit/vegetable consumption. This work exemplifies how BE features cascade through multiple behavioral mediators to affect cardiometabolic outcomes.

## 4 Vulnerable populations and environmental justice

Several other works investigate how BE impacts vary across population subgroups by age, gender, socioeconomic status, and migrant status, raising critical environmental justice concerns. The concentration of investigating older adults reflects both demographic imperatives and recognition that elderly populations experience heightened environmental sensitivity due to declining physical function and constrained activity spaces. Feng and Zheng document that older women's wellbeing responds more strongly to BE quality than men's, likely reflecting their greater domestic responsibilities, more localized activity patterns, and differential physiological sensitivities. This finding challenges gender-blind planning approaches and calls for designing environments attuned to gender needs. Meanwhile, He et al.'s study on urban village tenants and migrants highlights how informal housing serves as the primary shelter for low-income groups excluded from formal housing markets. Despite substandard conditions, these settlements foster community attachment that buffers mental health impacts, suggesting that social belonging may partially compensate for physical environmental deficits. Gu et al. note that older urban residents in areas undergoing renewal face compounded vulnerabilities, such as aging infrastructure, limited healthcare access, and economic precarity converge to elevate hypertension risk. The result shows these social determinants operate alongside and interact with BE exposures, underscoring the need for integrated interventions addressing both physical infrastructure and social services. Collectively, these findings demonstrate that environmental justice requires attending not only to distribution of resources (parks, transit, services) but also to how environmental effects vary by social position and how cumulative disadvantages multiply health risks.

## 5 From linear assumptions to non-linear realities

A methodological contribution throughout this Research Topic is the application of non-linear modeling techniques, which helps to capture threshold effects, diminishing returns, and interaction effects often obscured by conventional linear regression. For instance, Peng et al. adopt the GBDT model and SHAP value, which reveal that most BE variables exhibit non-monotonic relationships with metro usage, with effective ranges beyond which additional environmental improvements yield minimal behavioral change. Similarly, Song et al. document that cold island supply exhibits an inverted-U relationship with BE intensity, and it shows that moderate densification enhances cooling services, whereas excessive development degrades environmental quality despite increased green space investment. Gu et al. find that higher road network density increases hypertension risk by extending walking time and reducing fruit/vegetable consumption, which counter to the assumption that walkability universally benefits health. The non-linear reality demands nuanced, context-sensitive design that balances competing objectives (e.g., density for transit ridership vs. greenness for thermal comfort) and recognizes that more is not always better. These findings have profound planning implications. Rather than maximizing single environmental attributes, planners should calibrate interventions to optimal ranges while considering interactive and context-dependent effects.

## 6 Future directions

While the collections in this Research Topic advances the understanding of multi-scale environment-health relationships, several priorities remain for further research. First, most studies employ the cross-sectional design, which has limitation for the causal inference. It's helpful to employ the longitudinal and quasi-experimental designs that track environmental changes and health trajectories. Second, integrating GPS mobility data to activity-space exposure assessments could better capture dynamic environmental exposures beyond residential neighborhoods, particularly for working-age populations. Third, integrating objective environmental measures with subjective perceptions would illuminate how individual differences in environmental experience mediate health effects. Fourth, climate change may introduce compounding stressors, such as heat extremes, flood risks, and air pollution, as calls for conducting research on multi-hazard exposures and adaptation strategies. Finally, participatory approaches engaging communities in defining environmental priorities and co-designing interventions could ensure research translates to contextually appropriate, equitable solutions.

## 7 Conclusion

It's necessary to remind the importance of integrating multi-scale thinking, attention to behavioral and social pathways, commitment to environmental justice, and acknowledgment of non-linear complexities when creating health-promoting cities. As urbanization continues reshaping human habitats worldwide, evidence-based urban planning informed by rigorous environment-health research becomes ever more essential. We hope the collections in this Research Topic inspires scholars, practitioners, and policymakers to advance approaches that foster healthier, more equitable, and more sustainable urban futures for urban residents.

